# Dementia and the Impact of Acetylcholinesterase Inhibitors on Falls, Fractures and Mortality in a Geriatric Cohort: A 4-Year Follow-Up Study

**DOI:** 10.3390/jcm15145390

**Published:** 2026-07-09

**Authors:** Charles Inderjeeth, Diren Che Inderjeeth, Sneha Bharadwaj, Dani Kostova, Amanda Tillman, Angela Mei, Maxine Isbel

**Affiliations:** 1 Geriatric, Acute and Rehabilitation Medicine (GARM), Sir Charles Gairdner Osborne Park Health Care Group, Hospital Avenue, Perth, WA 6009, Australiaangela.mei@health.wa.gov.au (A.M.);; 2 School of Medicine, University of Western Australia, Crawley, Perth, WA 6009, Australia; 3 Fremantle Hospital, South Terrace, Perth, WA 6160, Australia

**Keywords:** dementia, osteoporosis, acetylcholinesterase inhibitors, mortality, fractures

## Abstract

**Objectives:** Dementia and osteoporosis are common and debilitating conditions that often coexist in older adults. We investigated mortality in patients with and without dementia as the primary outcome in a prospective memory clinic cohort. Falls and fractures were assessed as secondary outcomes, and associations with baseline acetylcholinesterase inhibitor (AChEI) use were explored. **Methods:** In a prospective observational cohort study, data were collected during routine clinical visits over four years. Data included demographics, dementia diagnosis, AChEI use, falls, fractures, bone mineral density (BMD) when clinically available, and mortality. Analysis included chi-square tests, Kaplan–Meier survival curves, Cox proportional hazards models, and recurrent-event models. Because AChEI analyses were exploratory and included several related endpoints, Benjamini–Hochberg false discovery rate (FDR) correction was applied to endpoint-level AChEI *p*-values. **Results:** 744 patients were enrolled; the mean age was 80.99 ± 6.8 years; 58.5% female; 55.8% with dementia. AChEI use was recorded in 113 patients (15.2%) at baseline. Over 4 years, 16.61% of participants experienced at least one fall with a cumulative fracture risk of 30.90%. Mortality was significantly higher in dementia patients (44.58% vs. 27.05%; *p* < 0.001). Dementia patients had double the mortality risk (OR: 1.956; 95% CI: 1.425–2.686). Annual and cumulative mortality rates increased progressively from 5.51% and in year 1 to 17.69% and 36.83% respectively by year 4. Baseline AChEI use was not significantly associated with mortality. Risk/100 for patient with dementia vs. without dementia for falls was 4.04 vs. 4.32 and fracture was 4.37 vs. 3.01. AChEI users had a trend of lower incidence/100 patient-years for falls of 3.01 vs. 4.37 and fractures 6.78 vs. 10.09. **Conclusions:** Dementia patients have higher mortality risk but not falls or fracture risk in this cohort. Although clinical cohort and animal studies suggest a benefit for AChEIs, this was not evident in this study possibly due to clinical cohort limitations. The trend of reduced falls and fracture in the AChEI cohort warrants further study.

## 1. Introduction

Dementia and osteoporosis are two common and debilitating conditions that often coexist in older adults, contributing substantially to morbidity and healthcare burden [1,2]. Australian epidemiological data estimate that in a population of 26 million, approximately 425,000 people are living with dementia. Of these, approximately 35 to 40 percent use anti-dementia medications such as acetylcholinesterase inhibitors (AChEIs) [3]. Osteoporosis in patients with dementia is associated with high morbidity and mortality [4] but is less likely to be treated [5]. Previous research has shown that low bone mineral density (BMD) and osteoporosis are related to an increased risk of Alzheimer’s disease [6,7].

Cholinergic activity is closely associated with bone health [8,9]. Patients with poliomyelitis have impaired bone growth in the affected limbs and an increased tendency to develop osteoporosis even when muscle activity recovers [10]. Botulinum neurotoxin causes transient hindlimb muscle paralysis by inhibiting the release of acetylcholine (ACh) from motor neurons and causes rapid bone degradation that does not fully recover after muscle function is restored [11,12]. However, the mechanism remains unclear.

ACh is released to relay neurologic information from one nerve to another. ACh is degraded by acetylcholinesterase, which stops nerve excitation after neurologic information has been relayed. Reduced levels of ACh have been linked with Alzheimer’s disease symptoms. Reversible AChEIs, including donepezil, rivastigmine, and galantamine, are used to manage the symptoms of Alzheimer’s disease by inhibiting the breakdown of ACh in the brain, thus enhancing cholinergic transmission [13,14].

A cohort study by Tamimi and colleagues in 2012 reported that treatment of Alzheimer’s disease with AChEIs was associated with lower rates of hip fracture in older patients [15]. The mechanism is uncertain. In a study published by our group, in vitro and in vivo mouse models suggested that AChEIs may improve bone biomarkers and quality by attenuating osteoclastogenesis and bone resorption [16]. Falls may also contribute to fracture risk in this population, although the relationship between AChEI exposure, falls, fractures, and survival remains uncertain in routine clinical cohorts.

This study examined a prospective cohort of older adults attending memory assessment clinics. The primary outcome was all-cause mortality by dementia status over four years. Secondary outcomes were falls and fractures by dementia status. Associations between baseline AChEI use and mortality, falls, and fractures were exploratory because AChEI treatment was clinically determined and not randomly assigned.

## 2. Materials and Methods

### 2.1. Study Design

This prospective observational cohort study recruited patients over three years from March 2016 to March 2019 and followed them for four years or until death at three metropolitan hospital memory assessment clinics in Perth, Western Australia. The study followed patients receiving routine clinical visits and care, without any intervention beyond standard care.

### 2.2. Participants

A total of approximately 1200 patients were registered for memory clinic attendance over the 3 years of recruitment from March 2016 to March 2019. The study recruited a cohort of 744 patients, aged 65 years and older, from 3 memory clinics across the three hospital sites. This was a convenient sample of consecutive patients reviewed in Dementia Assessment Clinics who agreed to be included in the study data collection. Patients were followed up for up to 4 years, loss to follow up or death. Participants were classified by dementia status. Overall, 415 (55.8%) had a confirmed dementia diagnosis and 329 (44.2%) did not have dementia. Alzheimer’s disease was the most common subtype, diagnosed in 321 participants (43.1% of the total cohort and 77.3% of those with dementia). AChEI treatment was recorded in 113 participants (15.2%) at baseline. AChEI treatment was prescribed as part of routine clinical care and was not randomly assigned.

### 2.3. Inclusion Criteria

Patients aged 65 years or older attending memory clinics were eligible. Patients with and without dementia diagnosis were included. Patients diagnosed with, or at risk of, osteoporosis were identified through clinical assessment or BMD testing when available.

### 2.4. Exclusion Criteria

Patients with severe cognitive impairment or advanced dementia that precluded reliable participation in follow-up were excluded. Patients or legal guardians could opt out of participation in the study.

### 2.5. Data Collection

Data were collected during routine clinical visits at baseline and at annual follow-up intervals over the four-year study period. Information collected included patient demographics, dementia diagnosis, AChEI medication use, BMD measurements when clinically available, falls, fractures, and mortality. Baseline routine-care fields also included MMSE where available, height, weight, BMI, osteoporosis risk screening variables, dementia medications, and selected bone-health medications including calcium, vitamin D, bisphosphonates, denosumab, selective estrogen receptor modulators, and hormone replacement therapy. These variables were not collected as complete protocolized longitudinal measures.

Because this was a usual-care memory clinic cohort rather than a protocolized osteoporosis or falls trial, availability of fall, fracture, cognitive, medication, and BMD data depended on clinic attendance, clinical documentation, patient or carer recall, and availability of hospital records. Mortality data were available for the full cohort. Falls and fracture data were recorded from annual clinical review and hospital records where available. BMD testing was clinically requested and was not mandated by the study protocol.

### 2.6. Falls

The number of falls in the previous year was recorded at each visit, with falls categorized as 0, 1 to 2, and more than 2 falls.

### 2.7. Fractures

The incidence of fractures, particularly hip fractures, was recorded annually. Fractures were categorized by type, including hip, rib, spine, and femur.

### 2.8. Bone Mineral Density

BMD was measured using dual-energy X-ray absorptiometry (DXA) at baseline and at follow-up intervals when clinically requested. Available BMD results were classified as normal, osteopenia, or osteoporosis based on World Health Organization criteria.

### 2.9. Mortality

Mortality data were collected annually and categorized by cause where available. Mortality rates were compared between patients with and without dementia.

### 2.10. Outcome Framework

The primary outcome was all-cause mortality by dementia status over four years. Secondary outcomes were falls and fractures by dementia status, including patient-level incidence and event-based incidence where evaluable follow-up was available. Exploratory outcomes were associations between baseline AChEI use and mortality, falls, and fractures. This hierarchy was used to guide the statistical analysis and interpretation.

### 2.11. Statistical Analysis

We used AI to help us build internal software tools. We used a proprietary, in-house AI model as a coding assistant to help write parts of custom Python scripts (this AI model was trained exclusively on our own internal data, which we have full rights to use). These scripts performed two specific tasks to ensure accuracy and consistency.

Baseline demographic data, dementia status, medication use, and clinical variables were summarized using means and standard deviations for continuous variables and frequencies and percentages for categorical variables. Mortality over the four-year period was compared between dementia and non-dementia patients using chi-square tests and Kaplan–Meier survival curves. Cox proportional hazards models were used for adjusted time-to-event analysis, including mortality and first-fracture analyses. Models adjusted for baseline dementia diagnosis, sex, and age.

Outcome-specific person-time was calculated from annual follow-up intervals with recorded fall or fracture status. For person-time analyses, follow-up for each outcome was censored at death, loss to follow-up, the first missing annual observation for that outcome, or the end of study. Missing annual fall and fracture intervals were not imputed. Patient-level incidence rates counted each participant once if they had at least one fall or fracture during evaluable follow-up. Event-based incidence rates counted all recorded fall or fracture events within evaluable intervals, including recurrent events in the same participant.

Recurrent falls were modeled with both Andersen–Gill Cox models and a Poisson log-linear model with log person-years as an offset and cluster-robust standard errors. These models were used because falls could occur more than once in the same participant and because evaluable follow-up time varied between participants. Cumulative fracture outcomes and AChEI group comparisons were summarized using odds ratios and 95% confidence intervals. BMD, GARVAN, and FRAX results were summarized descriptively where available.

Propensity score methods were considered for AChEI exposure. However, AChEI prescribing was only weakly explained by the available baseline variables, and several clinically relevant determinants of treatment allocation were not available in a complete standardized form for analysis. These included formal dementia severity characterization beyond available cognitive screening, functional status, frailty, mobility, medication tolerance, and prescribing context. In addition, baseline BMD was available only for a small clinically selected subset. Propensity score matching was therefore not used as the primary analytic approach, as it would have reduced the analytic sample without adequately addressing confounding by indication.

Because AChEI analyses were exploratory and the exposed group was small, Benjamini–Hochberg FDR correction was applied to endpoint-level AChEI *p*-values for mortality, falls, and fractures. The adjusted Cox model *p*-value was used for mortality, the Andersen–Gill recurrent-event *p*-value was used for falls, and the adjusted first-fracture Cox model *p*-value was used for fractures. The Poisson fall model and unadjusted fracture comparison were treated as supporting analyses and were not counted as separate endpoints for FDR correction. FDR-adjusted q-values were used to guide interpretation of AChEI associations. The primary mortality analysis by dementia status was not included in this exploratory AChEI correction family.

### 2.12. Ethical Considerations

Ethical approval for the study was obtained from the Central Human Research Ethics Committee. The Ethics Committee approved the study as not requiring written consent as data were collected as part of routine clinical care with no potential harm or experimental intervention. Patients and legal guardians were provided with an explanatory information sheet with the option to opt out of participation in the prospective study analysis. The study was conducted in accordance with the Declaration of Helsinki and Good Clinical Practice guidelines.

## 3. Results

A total of 744 patients were enrolled in the study, with a mean age of 80.99 ± 6.8 years. The cohort was 58.5% female (435) and 41.5% male (309). Dementia was diagnosed in 55.8% (415) of patients, while 44.2% (329) did not have dementia. Among those with dementia, Alzheimer’s disease was the most common subtype. Baseline cognitive screening and selected medication variables were captured in routine care where available, but these fields were not consistently available during follow-up.

BMD status at baseline was available for 11% of the cohort (82 of 744). As the main purpose of the clinic was cognitive assessment, clinicians did not routinely request BMD testing for all patients. Of those with available BMD data, 48% (39) had normal BMD, 17% (14) had osteoporosis, and 35% (29) had osteopenia. Mean T scores for femoral neck were −1.33 (SD = 1.3), total hip −0.93 (SD = 1.2), and lumbar spine −0.68 (SD = 1.7). The median 5-year GARVAN hip fracture risk was 2.0% (IQR: 0.9–7.0%), while the 10-year hip fracture risk was 5.0% (IQR: 2.0–14.0%). In patients with BMD data, the 5-year hip fracture risk was higher at 5.0% (IQR: 2.0–11.0%). For those assessed with the FRAX tool, the 10-year major osteoporotic fracture risk was 13.0% (IQR: 8.0–23.0%) without BMD and 19.0% (IQR: 11.0–27.0%) when BMD was included.

### 3.1. Falls and Fractures

Baseline falls data were available for 89.8% (668 of 744) of the cohort. At baseline, 65.87% (440 of 668 known cases) experienced no falls in the previous year, 23.80% (159 of 668 known cases) had 1 to 2 falls, and 10.33% (69 of 668 known cases) reported more than two falls. Falls data were missing for 10.22% (76 of 744 total cases). Baseline falls risk did not differ significantly across subgroups (Table 1).

By the end of the four-year period, falls data were available for 75.27% of the cohort (560 of 744). In this group, 83.39% (467 of 560 known cases) recorded no falls, and 16.61% (93 of 560 known cases) experienced at least one fall during the study period. Falls data were unavailable for 24.73% (184 of 744 total cases). Falls risk and cumulative falls risk did not differ significantly across subgroups.

**Figure 1:**
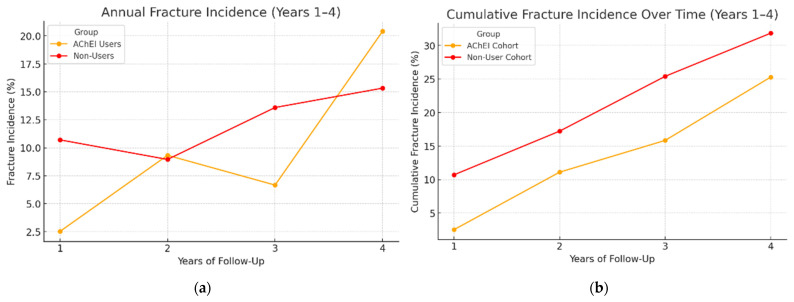
The annual and cumulative fracture incidence in AChEI users and non-users over four years. (**a**) Annual fracture incidence from year 1 to year 4, comparing participants using acetylcholinesterase inhibitors (AChEIs) with non-users. (**b**) Cumulative fracture incidence from year 1 to year 4, comparing AChEI users with non-users.

Fracture data were available for 576 patients. Spine fractures were reported in 10.07% (58 patients), hip fractures in 0.69% (4 patients), femur fractures in 7.29% (42 patients), and rib fractures in 2.26% (13 patients). The overall fracture rate increased during follow-up, with a rise from 9.52% (52 of 546 assessable cases) in year 1 to 16.07% (54 of 336 assessable cases) by year 4. By the end of the four-year period, 30.90% (178 of 576 known cases) of the cohort had sustained at least one fracture, while 69.10% (398 of 576 known cases) reported no fractures (Figure 1). Fracture data were missing or excluded for 22.58% (168 of 744 total cases).

### 3.2. Patient-Year Incidence Rates for Falls and Fractures

To account for varying follow-up times and outcome-specific missingness, incidence rates were reported using available patient-years for each endpoint. Two time-adjusted incidence measures were used. The first was a patient-level rate, in which each participant contributed no more than one fall or fracture outcome. In the full cohort, this rate was 4.16 patients with at least one fall per 100 patient-years and 9.57 patients with at least one fracture per 100 patient-years. In subgroup analyses, the corresponding patient-level fall rates were 4.32 and 4.02 per 100 patient-years in patients without and with dementia, respectively; patient-level fracture rates were 9.66 and 9.49 per 100 patient-years, respectively.

Observed unadjusted patient-level rates by AChEI use were 3.01 versus 4.37 per 100 patient-years for at least one fall, and 6.78 versus 10.09 per 100 patient-years for at least one fracture, in AChEI users and non-users respectively. These unadjusted rates should be interpreted alongside the adjusted models and with caution because AChEI exposure was clinically determined and not randomly assigned.

Falls were evaluable for 1568 patient-years. The recurrent-event rate was 5.1 falls per 100 patient-years. Neither the Andersen–Gill model nor the marginal Poisson model demonstrated a statistically significant association between baseline AChEI use and falls risk (aHR 0.78; 95% CI 0.42–1.44; *p* = 0.42; adjusted incidence-rate ratio [aIRR] 0.73; 95% CI 0.38–1.41; *p* = 0.35). The male sex showed lower recurrent fall estimates in both approaches (aHR 0.61; 95% CI 0.39–0.96; *p* = 0.034; aIRR 0.63; 95% CI 0.40–1.01; *p* = 0.056), and advancing age increased fall rates (aHR 1.05 per year; 95% CI 1.01–1.09; *p* = 0.008; aIRR 1.05 per year; 95% CI 1.02–1.09; *p* = 0.002).

**Figure 2:**
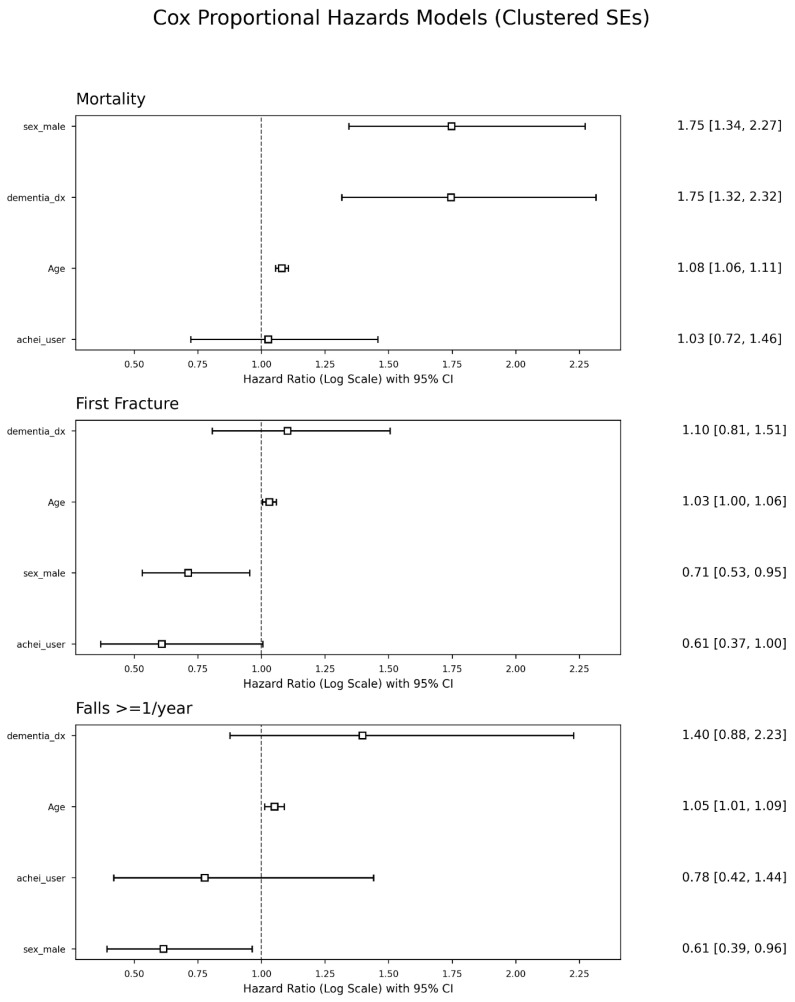
Hazard ratio for falls, fracture and mortality in the prospective patient cohort.

Fracture surveillance was restricted to intervals with available fracture data and yielded 1697 patient-years of observed follow-up. Across this evaluable follow-up, 269 fracture events were recorded, including recurrent fractures in the same participant, giving an event incidence rate of 15.8 fractures per 100 patient-years. For the separate adjusted first-fracture model shown in Figure 2, baseline AChEI exposure was not significantly associated with fracture hazard (aHR 0.61; 95% CI 0.37–1.00; *p* = 0.052). Male sex was associated with lower fracture hazard (aHR 0.71; 95% CI 0.53–0.95; *p* = 0.023), while older age increased fracture risk (aHR 1.03 per year; 95% CI 1.00–1.06; *p* = 0.025). Dementia diagnosis was not independently related to fracture hazard (aHR 1.10; 95% CI 0.81–1.51; *p* = 0.54).

### 3.3. Mortality

**Figure 3:**
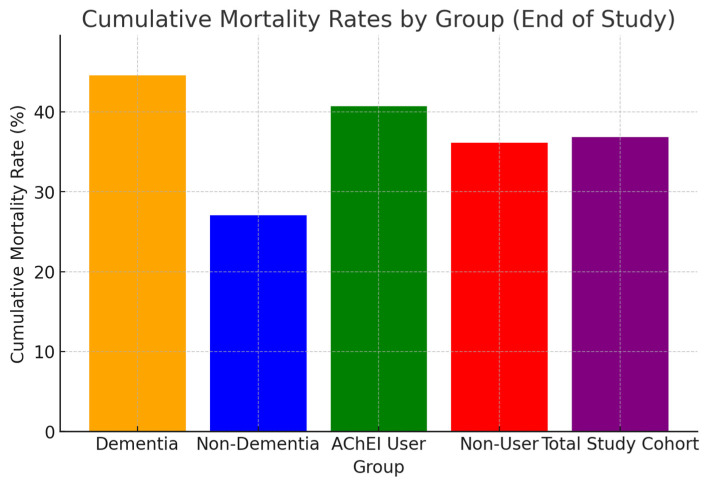
Cumulative mortality by subgroup in the prospective patient cohort, comparing the total cohort, AChEI-treated adults and non-AChEI-treated adults, and dementia vs. non-dementia groups. Note: AChEI = acetylcholinesterase inhibitor.

Mortality data were available for all patients in the cohort (Table 1, Figure 2 and Figure 3). By the end of the four-year period, 36.83% (274 of 744) of the total cohort had died. Mortality was significantly higher in patients with dementia, with deaths in 44.58% (185 of 415) compared with 27.05% (89 of 329) of non-dementia patients. This difference was statistically significant (*p* < 0.001), with dementia patients having nearly double the unadjusted mortality risk compared with non-dementia patients (OR: 1.956; 95% CI: 1.425–2.686) (Figure 2).

**Figure 4:**
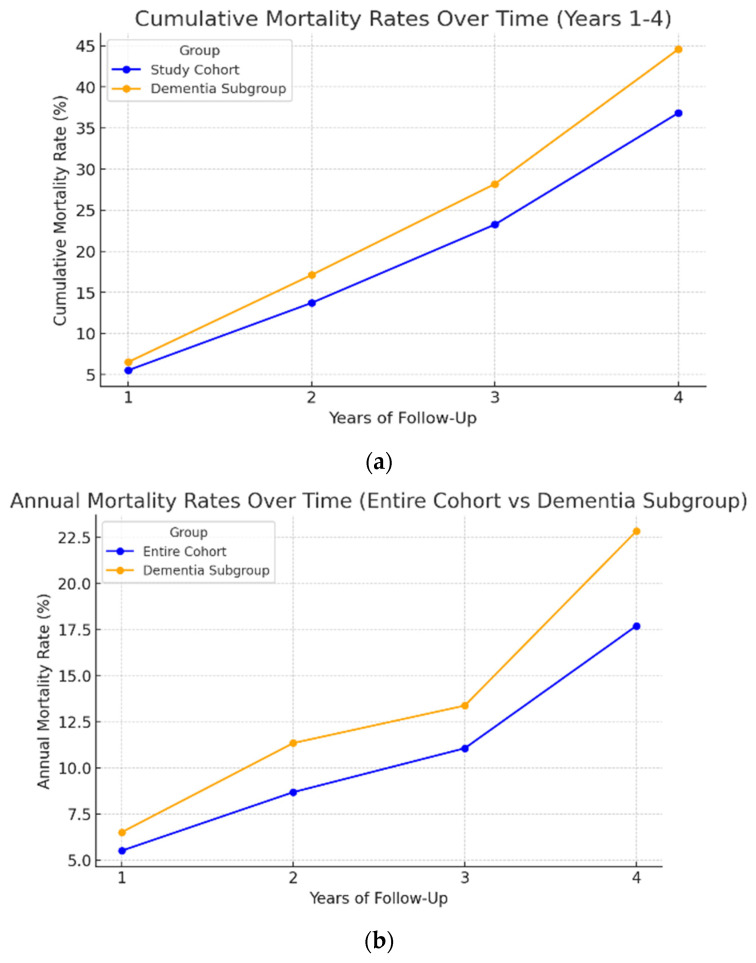
Annual and cumulative mortality in the total study cohort and dementia subgroup over four years. (**a**) Cumulative mortality rates from year 1 to year 4, comparing the total study cohort with participants diagnosed with dementia. (**b**) Annual mortality rates from year 1 to year 4, comparing the total study cohort with participants diagnosed with dementia.

Annual mortality rates increased from 5.51% (41 of 744) in year 1 to 8.68% (61 of 703) in year 2, 11.06% (71 of 642) in year 3, and 17.69% (101 of 571) by year 4. Consistent with this, cumulative mortality increased from 5.51% (41 of 744) in year 1 to 13.71% (102 of 744) in year 2, 23.25% (173 of 744) in year 3, and 36.83% (274 of 744) in year 4 (Figure 4).

### 3.4. Patient-Year Survival Analyses

For 2660 patient-years of follow-up in 744 participants, 274 deaths were recorded (10.3 deaths per 100 patient-years). Baseline AChEI exposure contributed 413 patient-years and was clustered in individuals with dementia. In the multivariable Cox model adjusting for age, sex, and baseline dementia diagnosis, AChEI use was not associated with mortality (aHR 1.03; 95% CI 0.72–1.46; *p* = 0.89). Dementia remained the dominant predictor of death (aHR 1.75; 95% CI 1.32–2.32; *p* < 0.001), and each increment in age conferred an 8% higher hazard (aHR 1.08; 95% CI 1.06–1.11; *p* < 0.001). Male sex was also associated with higher mortality (aHR 1.75; 95% CI 1.34–2.27; *p* < 0.001) (Figure 2).

### 3.5. Exploratory AChEI Analyses and FDR Correction

In univariate analysis, fracture occurrence over the 4-year period was 25.30% (21 of 83) in AChEI users and 31.85% (157 of 493) in non-users. This difference did not reach statistical significance (chi-square test = 1.425; *p* = 0.234). Observed unadjusted patient-level incidence rates were 3.01 versus 4.37 per 100 patient-years for falls and 6.78 versus 10.09 per 100 patient-years for fractures in AChEI users and non-users, respectively (Table 1 and Figure 2).

The unadjusted odds ratio for fracture occurrence in AChEI users compared with non-users was 0.7249 (95% CI: 0.4267–1.2314; *p* = 0.234). This estimate was interpreted as an exploratory comparison, with adjusted time-to-event and recurrent-event results reported separately. Over the 4-year study period, 14.63% (12 of 82 included) of AChEI users had at least one fall compared to 16.95% (81 of 478 included) of patients not using AChEIs. This difference in fall incidence was not statistically significant.

The mortality rate for AChEI users over the study was 40.71% (46 of 113), compared to 36.13% (228 of 631) in non-AChEI-users. This difference was not statistically significant, and AChEI treatment was not significantly associated with overall mortality (*p* = 0.353) (Figure 3).

After Benjamini–Hochberg FDR correction across the three endpoint-level AChEI analyses, no AChEI association remained statistically significant. The FDR-adjusted q-values were 0.156 for fracture hazard, 0.630 for recurrent falls using the Andersen–Gill model, and 0.890 for mortality using the Cox model. These results support interpreting AChEI analyses as exploratory and non-significant.

## 4. Discussion

This study examined mortality, falls, fractures, and baseline AChEI use in a real-world memory clinic cohort followed for four years. The primary finding was higher mortality in older adults with dementia compared with those without dementia. Falls and fractures were common, but dementia was not independently associated with fracture hazard in the adjusted analysis. AChEI analyses did not identify significant associations with mortality, falls, or fractures, and this remained the case after FDR correction.

The mortality finding is consistent with the broader geriatric literature. Dementia is associated with loss of physiological reserve, dependency, falls, multimorbidity, and higher mortality. In our cohort, 44.58% of patients with dementia died during follow-up compared with 27.05% of patients without dementia, and dementia remained associated with mortality after adjustment for age and sex. A recent systematic review of Alzheimer’s disease burden reported 5-year mortality of approximately 35% and higher mortality in Alzheimer’s disease than in age- and year-matched controls [17]. This aligns with clinical studies showing that cognitive impairment and dependence are important mortality predictors in older adults. Gomez-Ramos and colleagues, in a Mexican emergency department cohort, reported that cognitive impairment and dependence were independent predictors of in-hospital mortality, while frailty, falls risk, delirium, and pressure ulcer risk were also associated with mortality [18]. Although that population was an acute care cohort and differs from our memory clinic cohort, the findings support the relevance of geriatric syndromes when interpreting mortality risk in cognitively impaired older adults.

Other prospective cohort data also support a broad geriatric interpretation of mortality risk. In the ELSIA study from Brazil, mortality risk in older adults was associated with social, health, functional, and behavioral factors. The number of prescribed medications was associated with higher mortality, while greater physical activity and larger hip circumference were associated with lower mortality risk [19]. Our study did not include complete longitudinal measures of physical activity, nutritional status, frailty, or sarcopenia. This limits our ability to determine whether the mortality signal associated with dementia was mediated by frailty, poor nutrition, low muscle mass, immobility, polypharmacy, or other geriatric syndromes.

The role of sex differed by outcome. Male sex was associated with higher mortality in this cohort, which is consistent with many older adult survival studies. In contrast, male sex showed lower fracture hazard and lower recurrent fall estimates in the fall and fracture models. This should not be interpreted as a simple biological protective effect. Possible explanations include lower osteoporosis burden in men, different patterns of fall reporting, different fracture ascertainment, survival effects, or unmeasured differences in frailty, mobility, nutrition, and care exposure. Because formal frailty indices, functional status, mobility status, and comprehensive nutritional measures were not available in a complete standardized form, these findings require cautious interpretation.

Falls and fractures were frequent in this memory clinic cohort, but fracture risk was not significantly higher in patients with dementia compared with those without dementia. The patient-level incidence rate for at least one fracture was 9.57 per 100 patient-years, while the event-based fracture rate, including recurrent fractures in the same patient, was 15.8 per 100 patient-years. For falls, the corresponding patient-level and recurrent-event rates were 4.16 and 5.1 per 100 patient-years. Reporting both measures clarifies that the lower rates describe patients with at least one recorded event, while the higher rates describe the total recorded event burden. The annual fracture rate increased from year 1 to year 4, suggesting that fracture prevention remains clinically important during ongoing follow-up of older memory clinic patients.

The absence of a significant excess fracture risk in dementia differs from the higher fracture risk often reported in dementia populations, where cognitive impairment, balance problems, sarcopenia, vitamin D deficiency, and reduced physical activity may contribute to fracture risk [4,5,7]. Recent meta-analytic evidence also suggests that older adults with Alzheimer’s disease have lower BMD than older adults without dementia, particularly at the femoral neck [20]. Several factors may explain the difference in our cohort. Hip fractures were uncommon, BMD was available only for a clinically selected minority, and the frailest patients may have been under-represented because severe cognitive impairment limited participation in the follow-up. In addition, patients attending memory clinics may have received multidisciplinary care that reduced fall or fracture risk. Spine and femur fractures were more common than hip fractures in this cohort, raising the possibility that vertebral fragility and non-hip fractures are important outcomes in this population. Further work is required to determine whether vertebral fractures are under-recognized or under-reported in older adults with dementia.

### 4.1. Impact of AChEI Treatment

AChEI use was uncommon and clinically selected. Only 113 patients used AChEIs at baseline, representing 15.2% of the cohort, and outcome-specific evaluable denominators were smaller for falls and fractures because of missing follow-up data. This limited exposed sample increases the risk of type II error, particularly for fracture and hip fracture outcomes. AChEI treatment was also not randomly assigned. Prescribing may have reflected dementia subtype, cognitive profile, medication tolerance, contraindications, carer support, specialist review, and healthcare engagement. These factors could influence falls, fractures, and mortality independently of medication effect.

Prior studies have suggested possible associations between AChEI use and lower fracture risk [15,21], and our preclinical work suggested that AChEIs may influence bone metabolism through effects on osteoclastogenesis and bone resorption [16]. In this cohort, however, baseline AChEI use was not significantly associated with fracture risk, falls, or overall mortality. Observed unadjusted patient-level rates were lower in AChEI users than non-users for falls and fractures, but these differences were not statistically significant and should not be interpreted as evidence of reduced risk. The non-significant findings may reflect the small sample size of AChEI users, the low incidence of hip fracture, residual confounding by indication, or absence of a clinically meaningful association in this cohort. After FDR correction, the smallest endpoint-level AChEI q-value was 0.156. These results do not support a clinical protective effect in this cohort. They also do not exclude any smaller effects that this study was underpowered to detect.

The current evidence on AChEIs in older people with dementia requires balance. AChEIs may provide cognitive and functional benefits in selected patients, but adverse drug reactions are clinically important in older adults. Observational mortality studies of anti-dementia drug exposure are also difficult to interpret because treatment selection is linked to clinical status and care context [22]. Ruangritchankul and colleagues reviewed adverse drug reactions of AChEIs and noted gastrointestinal, neuropsychiatric, and cardiovascular adverse effects, including bradycardia, heart block, syncope, and outcomes such as falls, fractures, and hospitalization [23]. Medication burden is also relevant to falls in dementia. In a Korean self-controlled case series, potentially inappropriate medication use among older adults with dementia was associated with increased falls or fall-related injuries [24]. In this context, the absence of a significant association in our cohort should not be interpreted as evidence of musculoskeletal benefit or safety. Rather, AChEI prescribing should remain individualized, with attention to cognition, function, adverse effects, falls history, cardiovascular risk, and medication burden.

### 4.2. Strengths and Limitations

The study has several strengths. It was a prospective real-world cohort of older adults attending memory clinics, with four-year mortality follow-up and clinically relevant fall and fracture outcomes. It also examined a question of geriatric clinical relevance: whether dementia and AChEI exposure are associated with mortality and musculoskeletal outcomes in routine practice. The cohort reflects routine care rather than a highly selected trial population, which increases clinical relevance for memory clinic services.

However, missing data were an important limitation. Mortality was complete, but falls, fractures, BMD, cognitive screening, medication exposure, and osteoporosis risk variables were not complete longitudinal measures. Falls and fractures were collected in routine care and depended on clinic attendance, clinical documentation, patient or carer recall, and hospital record availability. BMD was clinically requested rather than protocol-mandated and was available for only 11% of participants. Missingness was therefore likely to be informative rather than completely random. Patients who missed follow-up, died, entered residential care, became too frail to attend, or received care outside the participating services may have differed systematically from those with complete data.

The observational design also precludes causal conclusions. Confounding by indication is particularly relevant for AChEI analyses, as treated patients may differ from untreated patients in dementia subtype, dementia severity, functional status, frailty, medication tolerance, access to specialist care, and overall healthcare engagement. There may also have been selection bias, with under-representation of the frailest and most vulnerable patients due to exclusion factors, patient or carer choice, and follow-up limitations. Alternatively, the lower-than-expected hip fracture rate may reflect the quality of multidisciplinary clinical care, including strategies to reduce falls and fractures. The study lacked complete longitudinal measures of dementia severity, frailty, mobility, function, nutrition, comorbidity burden, corticosteroid exposure, medication changes, AChEI dose, and AChEI duration. These limitations restrict the ability to adjust fully for confounding and to interpret AChEI associations.

### 4.3. Future Directions

Future studies should include larger samples of AChEI users, frailer populations, more complete BMD and fracture risk assessment, standardized dementia severity measures, functional and frailty assessment, nutritional markers, medication exposure over time, and longer follow-up. Such studies would be better placed to determine whether AChEIs have clinically meaningful effects on falls, fractures, and survival in older adults with dementia. Studies that include higher hip fracture risk populations may also clarify whether the associations reported in previous hip fracture studies apply to broader memory clinic cohorts. Randomized controlled studies would provide the strongest evidence but may be difficult to conduct in this older and clinically complex population. Further evaluation of mechanisms linking dementia with bone fragility, including vitamin D deficiency, immobility, falls, sarcopenia, and changes in muscle strength, may help identify targeted strategies to reduce fracture risk.

## 5. Conclusions

In this prospective memory clinic cohort, dementia was associated with higher four-year mortality. Fall and fracture rates were clinically important, but fracture risk was not significantly different between dementia and non-dementia patients, and dementia diagnosis was not independently associated with fracture hazard in the adjusted analysis. Baseline AChEI use was uncommon and was not significantly associated with mortality, falls, or fractures. After endpoint-level FDR correction across AChEI analyses, no AChEI association remained statistically significant. These findings should be interpreted as exploratory and do not establish benefit or harm of AChEI treatment for falls, fractures, or mortality.

The results support comprehensive geriatric care for older adults with dementia, including attention to cognition, frailty, falls, bone health, nutrition, medication burden, and survival risk. Further research is needed to assess whether AChEIs have clinically meaningful effects on musculoskeletal health and fracture risk in patients with dementia.

## Figures and Tables

**Table 1:** Outcomes of clinic patients included in the prospective cohort study.

Outcome	Baseline Total (n = 744)	Year 1	Year 2	Year 3	Year 4	Cumulative *	Univariate Analysis	Unadjusted OR (95% CI)
**Falls**	34.13% (228/668)					16.61% (93/560)		
**Mortality in Dementia**		6.51% (27/415)	11.34% (44/388)	13.37% (46/344)	22.82% (68/298)	44.58% (185/415)	*p* < 0.001	1.956 (1.425–2.686)
**Mortality in Non-Dementia**		4.26% (14/329)	5.4% (17/315)	8.39% (25/298)	12.09% (33/273)	27.05% (89/329)		
**Fracture in Dementia**	22.68% (83/366)	10.70% (32/299)	7.81% (20/256)	12.38% (26/210)	16.86% (29/172)	30.70% (97/316)	*p* = 0.078	
**Fracture Non-Dementia**	23.65% (70/296)	8.10% (20/247)	10.34% (24/232)	12.81% (26/203)	15.24% (25/164)	31.15% (81/260)		
**Fracture incidence in AChEI users**		2.53% (2/79)	9.33% (7/75)	6.67% (4/60)	20.41% (10/49)	25.30% (21/83)	*p* = 0.234	0.724 (0.426–1.231)

Note: AChEI = acetylcholinesterase inhibitor. The mortality *p*-value compares the paired dementia and non-dementia mortality rows. The dementia/non-dementia fracture *p*-value compares the paired fracture rows. The AChEI fracture *p*-value and unadjusted OR compare AChEI users with non-users (21/83 vs. 157/493). The falls row is descriptive for the cohort and does not represent a between-group comparison. * Patient-level incidence rates count each participant only once if they experienced at least one fall or sustained at least one fracture during evaluable follow-up.

## Data Availability

The data presented in this study are available on request from the corresponding author. This study is based on data collected during clinical care. It was not part of the approved application at the time of ethics application. However, the data may be shared for scientific purposes with ethics committee approval.

## Abbreviations

The following abbreviations are used in this manuscript:

ACh	acetylcholine
AChEI(s)	acetylcholinesterase inhibitor(s)
aHR	adjusted hazard ratio
aIRR	adjusted incidence-rate ratio
BMD	bone mineral density
BMI	body mass index
CI	confidence interval
DXA	dual-energy X-ray absorptiometry
FDR	false discovery rate
FRAX	Fracture Risk Assessment Tool
GARVAN	Garvan fracture risk calculator
IQR	interquartile range
MMSE	Mini-Mental State Examination
OR	odds ratio
SD	standard deviation
